# Association between carotid atherosclerotic plaque characteristics and white matter hyperintensity in cerebral small vessel disease

**DOI:** 10.3389/fneur.2025.1745554

**Published:** 2026-01-09

**Authors:** Qiuhao Feng, Tianle Wang, Li Zhu

**Affiliations:** Department of Radiology, Affiliated Hospital 2 of Nantong University, Nantong, China

**Keywords:** carotid atherosclerosis, cerebral small vessel disease, CTA, plaque thickness, WMH

## Abstract

**Objective:**

This study aimed to investigate the relationship between carotid atherosclerotic plaque characteristics, as assessed by computed tomography angiography (CTA), and white matter hyperintensity (WMH) in patients with carotid atherosclerosis.

**Methods:**

A total of 180 patients with carotid atherosclerosis who underwent both carotid CTA and brain MRI were retrospectively enrolled. WMH severity on T2-weighted fluid-attenuated inversion recovery (T2-FLAIR) images was graded using the Fazekas scale, with patients categorized into the none/mild (*n* = 77) and severe (*n* = 103) WMH groups. Plaque thickness, length, and stenosis were measured on CTA multiplanar reconstruction (MPR) and curved planar reformation (CPR) images. Plaque calcification types were classified according to Saba’s criteria. Quantitative WMH volumes were automatically segmented into four subregions using an artificial intelligence-assisted platform. Logistic regression and Spearman correlation analyses were performed to evaluate associations between plaque characteristics and WMH burden.

**Results:**

Patients with severe WMH exhibited greater plaque thickness (4.20 vs. 2.80 mm, *p* < 0.001) and higher stenosis (52.0% vs. 28.0%, *p* < 0.001). After multivariate adjustment using a fully adjusted model including demographic variables, vascular risk factors, and lipid profile parameters, maximal plaque thickness (OR = 1.669) and stenosis degree (OR = 1.044) remained independent predictors of severe WMH (*p* < 0.001). Plaque thickness showed the strongest correlations with WMH volumes in the periventricular (*r* = 0.429) and juxtaventricular (*r* = 0.383) regions (*p* < 0.001), whereas plaque length showed no significant associations. Rim sign-positive calcified plaques (type 6) were significantly correlated with total WMH volume (*β* = 0.359, *p* = 0.045).

**Conclusion:**

Carotid plaque thickness and stenosis are independently associated with WMH severity, particularly affecting periventricular regions. These findings suggest that carotid atherosclerosis may contribute to cerebral small vessel disease (CSVD) progression through hemodynamic or embolic mechanisms.

## 1 Introduction

Cerebral small vessel disease (CSVD) is increasingly recognized as one of the most prevalent pathological processes affecting the aging brain and plays a crucial role in the development of stroke and cognitive decline (1). Neuroimaging serves as the cornerstone for CSVD diagnosis, with magnetic resonance imaging (MRI) being the most sensitive modality. Among the characteristic MRI manifestations, white matter hyperintensity (WMH) is the most common imaging biomarker of CSVD (2, 3). WMH typically appears as hyperintense lesions in deep and periventricular white matter on T2-weighted or fluid-attenuated inversion recovery (FLAIR) sequences, usually exhibiting symmetrical distribution.

Emerging evidence suggests that CSVD and large-artery atherosclerosis may share overlapping risk factors such as hypertension, diabetes, dyslipidemia, and advanced age (4–6). With advancing age, the risk of ischemic stroke increases dramatically (2, 7, 8). Therefore, exploring the potential interplay between carotid atherosclerosis and CSVD has become increasingly important for understanding cerebrovascular pathology and improving preventive strategies.

Carotid atherosclerosis, as a manifestation of systemic vascular disease, may contribute to WMH formation through several mechanisms. These include chronic cerebral hypoperfusion secondary to arterial narrowing, microembolization from unstable plaques, and endothelial dysfunction mediated by inflammatory and hemodynamic factors. Recent studies have demonstrated associations between extracranial carotid plaque characteristics and WMH severity (2, 9, 10); however, most previous studies relied on ultrasound-based measurements, which have limited sensitivity for plaque composition and calcification morphology.

Computed tomography angiography (CTA) enables high-resolution visualization of carotid plaque morphology, including the degree of stenosis, plaque thickness, and calcification pattern. The advancement of artificial intelligence-assisted image processing has further improved the accuracy and reproducibility of WMH quantification. Nonetheless, few studies have systematically examined the association between CTA-based carotid plaque features and quantitative WMH volumes across distinct white matter subregions.

Therefore, this study aimed to (1) evaluate the independent association between carotid plaque morphology and WMH severity using CTA and MRI, (2) analyze the spatial relationship between plaque characteristics and regional WMH volumes, and (3) explore the influence of different calcification subtypes on WMH burden. We hypothesized that greater plaque thickness and higher carotid stenosis would be independently associated with severe WMH, particularly in periventricular regions, and that rim sign-positive calcifications might indicate a higher risk of CSVD progression.

## 2 Materials and methods

### 2.1 Study population

Between January 2024 and January 2025, a total of 192 hospitalized patients diagnosed with carotid atherosclerosis at the Affiliated Hospital 2 of Nantong University were retrospectively screened. After excluding 12 patients (7 due to poor CTA image quality and 5 who did not undergo MRI), 180 patients were included in the final analysis (128 males and 52 females; mean age: 71 years). Inclusion criteria were: (1) age ≥18 years, (2) CTA-confirmed carotid atherosclerotic plaque, and (3) brain MRI performed within 1 week of CTA. Exclusion criteria were: (1) acute cerebral infarction with confirmed occlusion of carotid or intracranial major arteries (circle of Willis and main branches); (2) WMH caused by other etiologies such as multiple sclerosis, toxic/metabolic encephalopathy, or hereditary small vessel disease; (3) prior carotid stenting or endarterectomy; and (4) inadequate imaging quality.

### 2.2 Clinical and laboratory data

Baseline characteristics were collected, including age, sex, smoking and drinking status, and comorbidities such as hypertension, diabetes, and hyperlipidemia. Laboratory parameters included triglycerides (TG), total cholesterol (TC), high-density lipoprotein (HDL-C), and low-density lipoprotein (LDL-C) levels.

### 2.3 CTA acquisition and plaque analysis

All patients underwent head and neck CTA using a third-generation dual-source CT scanner (SOMATOM Force, Siemens Healthcare, Forchheim, Germany). Patients were scanned in the supine position, with contrast medium (iodine concentration 370 mg/mL, 30–40 mL) injected via the antecubital vein at a rate of 4–5 mL/s. The scanning range covered from the aortic arch to the skull base, with parameters as follows: Sn150 kV and 90 kV tube voltages, 100–180 mAs and 130–330 mAs tube currents, rotation time 0.28 s/r, slice thickness 0.75 mm, and interslice spacing 0.5 mm.

The acquired images were transferred to a post-processing workstation (*syngo*.via, Siemens Healthcare) for multiplanar reconstruction (MPR) and curved planar reformation (CPR). Plaque thickness (mm), plaque length (mm), and luminal stenosis (%) were measured. Each measurement was performed three times, and the average was used as the final result. The maximum plaque thickness and length were determined from both sides. The degree of stenosis was calculated using the North American Symptomatic Carotid Endarterectomy Trial (NASCET) formula (11):


$$\text{Stenosis}(\%)=(\frac{\begin{array}{l} \text{Normal distal diameter}\\ -\text{Residual lumen}\;\;\text{diameter} \end{array}}{\text{Normal distal diameter}})\times 100\%$$

Plaque calcification was categorized into six subtypes according to Saba et al. (12):

Type 1, non-calcified.
Type 2, intimal/superficial calcification.
Type 3, large irregular calcification.
Type 4, rim sign-negative.
Type 5, mixed intimal and bulky calcification.
Type 6, rim sign-positive (defined as adventitial calcification with >2 mm intraplaque soft component).

### 2.4 MRI acquisition and WMH quantification

**Figure 1:**
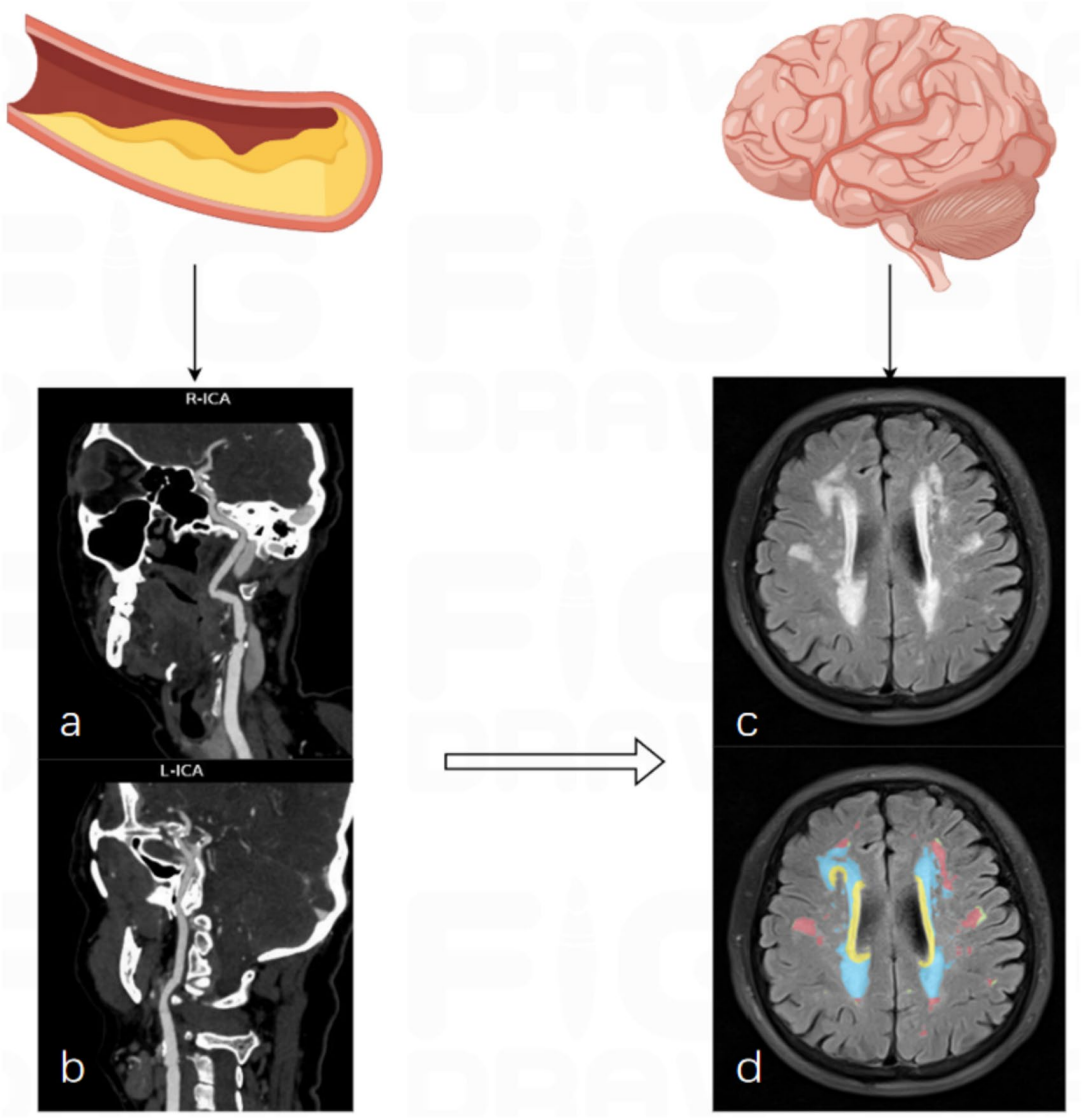
MRI and CTA images in a patient with CSVD. **(a,b)** CTA images showing carotid atherosclerotic plaques at the carotid bifurcation. **(c)** FLAIR image demonstrating severe periventricular and deep WMH. **(d)** Automated segmentation of WMH subregions using the uAI Discover platform.

MRI sequences included T1-weighted imaging, T2-weighted imaging, T2-FLAIR, and diffusion-weighted imaging (DWI). WMH was identified and quantified using the uAI Discover CSVD v2.0 platform (United Imaging Healthcare, Shanghai, China). The uAI Discover CSVD platform applies a deep learning-based convolutional neural network trained on expert-annotated datasets to automatically identify and segment WMH on T2-FLAIR images. The algorithm incorporates anatomical priors and intensity-based constraints to reduce misclassification of perivascular spaces, lacunes, and cerebrospinal fluid. WMH was quantitatively segmented into four subregions: periventricular, juxtaventricular, deep, and subcortical white matter (Figure 1). All automated WMH segmentation results were independently reviewed by two experienced radiologists blinded to clinical data. Obvious segmentation errors were manually corrected when necessary.

WMH severity was scored according to the Fazekas scale (13). A total Fazekas score ≥3 was defined as severe WMH, while a score <3 was classified as none/mild WMH (14). All imaging measurements were independently performed by two trained radiologists blinded to clinical data. Interobserver agreement was assessed using the intraclass correlation coefficient (ICC), with ICC >0.8 indicating excellent consistency.

### 2.5 Statistical analysis

All statistical analyses were performed using SPSS 27.0 and R 4.5.1. Continuous variables were tested for normality using the Kolmogorov–Smirnov test. Normally distributed data were presented as mean ± SD and compared with the independent-sample *t*-test, while non-normally distributed data were expressed as median (Q1, Q3) and compared using the Mann–Whitney *U* test. Categorical variables were expressed as frequencies and compared using the *χ*^2^ test.

Univariate and multivariate logistic regression analyses were used to identify independent predictors of severe WMH. Three models were constructed: Model 1: adjusted for age and sex; Model 2: further adjusted for vascular risk factors (smoking, alcohol use, hypertension, diabetes, hyperlipidemia); Model 3 represented the fully adjusted model, additionally including lipid profile parameters (triglycerides, total cholesterol, HDL-C, and LDL-C).

**Figure 2:**
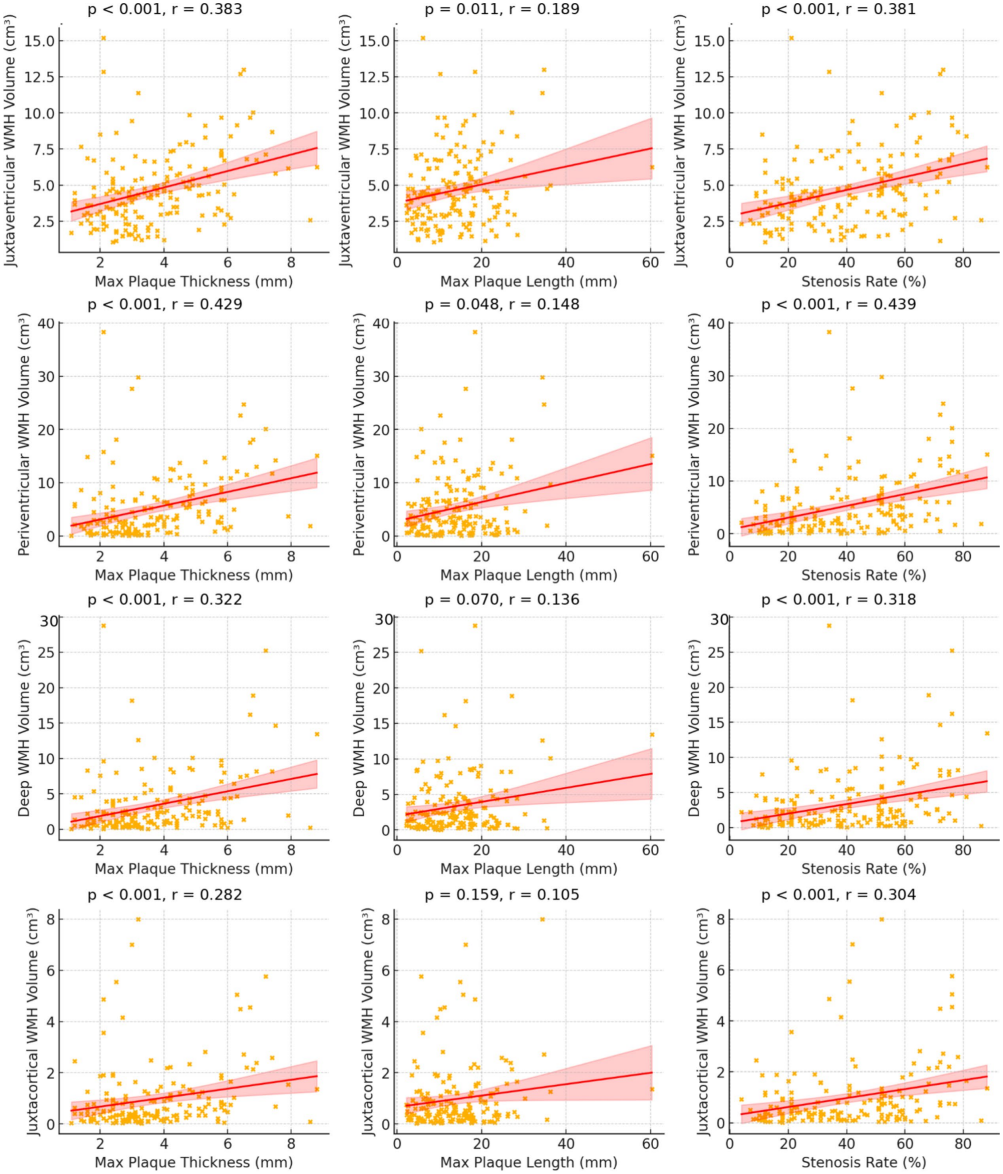
Scatter plots illustrating the relationships between carotid plaque features and regional WMH volumes. Each dot represents an individual patient. The fitted line and shaded area are shown for visualization of trends. Spearman correlation coefficients (*r*) and *p*-values are displayed in each panel. The complete set of Spearman *r* and *p*-values for all associations is also summarized in Table 3.

**Figure 3:**
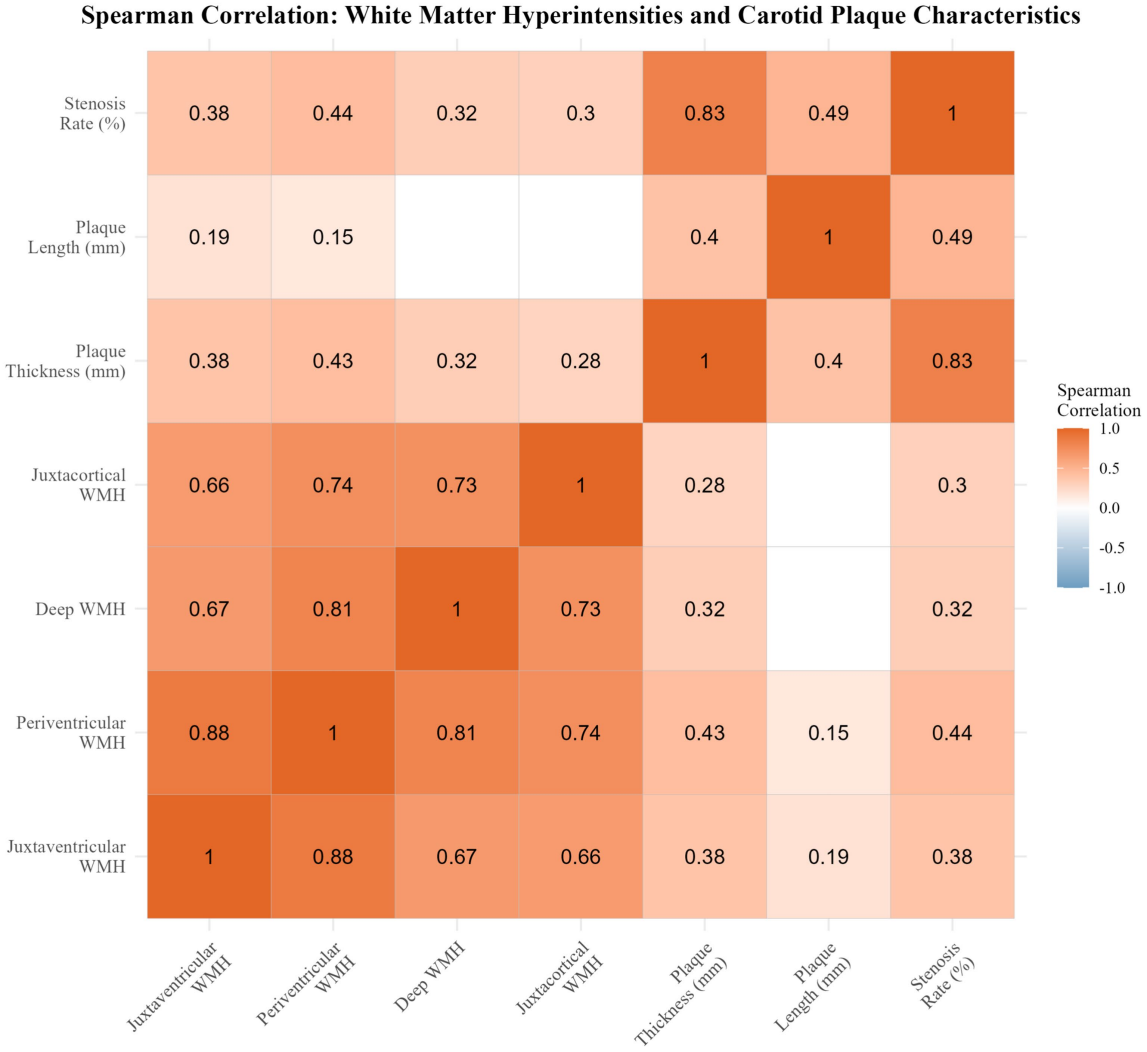
Correlation matrix of white matter hyperintensity volumes and carotid plaque characteristics.

Spearman correlation was performed to assess associations between plaque morphology (maximum thickness, length, stenosis) and WMH volumes in the four subregions. The results are visualized using scatter plots (Figure 2) and a correlation heatmap (Figure 3). A multiple linear regression model was applied to assess the impact of different calcification types (type 1 as reference) on total WMH volume, with adjustments for potential confounders (age, hypertension, hyperhomocysteinemia, plaque thickness, and stenosis). A *p*-value <0.05 was considered statistically significant.

## 3 Results

### 3.1 Baseline characteristics and plaque features

Among the 180 included patients, 77 had none/mild WMH and 103 had severe WMH. Patients in the severe WMH group were significantly older (median 73.0 vs. 64.0 years, *p* < 0.001). The prevalence of hyperlipidemia (35.0% vs. 57.1%, *p* = 0.005), TG (1.38 vs. 1.74 mmol/L, *p* = 0.005), and LDL-C levels (2.58 vs. 2.78 mmol/L, *p* = 0.033) were all lower in the severe WMH group—likely reflecting statin therapy in older patients.

The severe WMH group exhibited significantly greater plaque thickness (4.20 vs. 2.80 mm, *p* < 0.001) and higher stenosis (52.0% vs. 28.0%, *p* < 0.001). No significant differences were observed in sex, hypertension, diabetes, smoking, or plaque length between groups (*p* > 0.05) (Table 1).

**Table 1:** Baseline characteristics and plaque features in patients with different WMH severity levels.

Variables	None/Mild WMH (*n* = 77)	Severe WMH (*n* = 103)	*p*
General information			
Sex, male [*n* (%)]	54 (70.1)	74 (71.8)	0.932
Age (years)	64.00 (57.00, 72.00)	73.00 (67.50, 78.00)	<0.001
Hypertension [*n* (%)]	53 (68.8)	79 (76.7)	0.312
Diabetes [*n* (%)]	24 (31.2)	37 (35.9)	0.612
Hyperlipidemia [*n* (%)]	44 (57.1)	36 (35.0)	0.005
Smoking [*n* (%)]	38 (49.4)	44 (42.7)	0.464
Alcohol consumption [*n* (%)]	24 (31.2)	34 (33.0)	0.872
Laboratory indicators			
Total cholesterol (mmol/L)	4.34 (3.69, 5.13)	4.11 (3.41, 4.77)	0.176
Triglycerides (mmol/L)	1.74 (1.33, 2.22)	1.38 (1.02, 1.86)	0.005
HDL-C (mmol/L)	1.12 (0.93, 1.30)	1.09 (0.96, 1.29)	0.770
LDL-C (mmol/L)	2.78 (2.34, 3.52)	2.58 (2.00, 3.12)	0.033
Plaque characteristics			
Maximum plaque thickness (mm)	2.80 (2.20, 3.50)	4.20 (3.00, 5.35)	<0.001
Maximum plaque length (mm)	10.70 (6.90, 18.00)	13.50 (8.80, 17.50)	0.345
Stenosis degree (%)	28.00 (18.00, 38.00)	52.00 (32.00, 61.00)	<0.001

Continuous variables are presented as median (interquartile range), categorical variables as number (%).

Comparisons used Mann–Whitney *U* or chi-square tests as appropriate.

WMH, white matter hyperintensity; HDL-C, high-density lipoprotein cholesterol; LDL-C, low-density lipoprotein cholesterol.

### 3.2 Independent associations between plaque features and WMH

In univariate logistic regression, age (OR = 1.09, 95% CI: 1.05–1.13), maximum plaque thickness (OR = 1.76, 95% CI: 1.39–2.24), and stenosis (OR = 1.05, 95% CI: 1.03–1.06) were significantly associated with severe WMH. After multivariate adjustment using Model 3 (fully adjusted for lipid profile parameters), maximum plaque thickness (OR = 1.669, 95% CI: 1.288–2.218) and stenosis (OR = 1.044, 95% CI: 1.024–1.066) remained independent predictors of severe WMH (*p* < 0.001). Plaque length was not significantly associated with WMH severity across all models (Table 2).

**Table 2:** Multivariable logistic regression for predictors of severe WMH.

Variables	Model 1 OR (95% CI)	*p*	Model 2 OR (95% CI)	*p*	Model 3 OR (95% CI)	*p*
Maximum plaque thickness	1.555 (1.223–2.021)	<0.001	1.592 (1.246–2.078)	<0.001	1.669 (1.288–2.218)	<0.001
Maximum plaque length	1.014 (0.974–1.058)	0.506	1.026 (0.983–1.073)	0.250	1.027 (0.983–1.075)	0.247
Stenosis degree	1.038 (1.020–1.058)	<0.001	1.039 (1.021–1.060)	<0.001	1.044 (1.024–1.066)	<0.001

Model 1: adjusted for age and sex. Model 2: Model 1 + smoking, alcohol, hypertension, diabetes, hyperlipidemia. Model 3: Model 2 + triglycerides, total cholesterol, HDL-C, LDL-C.

### 3.3 Correlations between plaque morphology and regional WMH volumes

To facilitate interpretation of the Spearman correlation analyses, the relationships between carotid plaque morphology and regional WMH volumes are visualized using scatter plots (Figure 2) and a correlation heatmap (Figure 3). Spearman correlation analyses demonstrated that maximum plaque thickness had the strongest and most consistent positive correlations with WMH volumes across subregions, particularly periventricular (*r* = 0.429, *p* < 0.001) and juxtaventricular regions (*r* = 0.383, *p* < 0.001). Stenosis also showed moderate positive correlations with WMH volume in all regions (*r* = 0.304–0.439; all *p* < 0.001). In contrast, plaque length exhibited weaker correlations, reaching significance only in juxtaventricular (*r* = 0.189, *p* = 0.011) and periventricular regions (*r* = 0.148, *p* = 0.048). The corresponding Spearman correlation coefficients (*r*) and *p*-values for all plaque features and WMH subregions are summarized in Table 3.

**Table 3:** Correlation between plaque morphology and WMH volume in different subregions.

WMH region	Plaque feature	*r*	*p*	Correlation strength
Juxtaventricular WMH	Maximum thickness (mm)	0.383	<0.001	Moderate
	Maximum length (mm)	0.189	0.011	Weak
	Stenosis (%)	0.381	<0.001	Moderate
Periventricular WMH	Maximum thickness (mm)	0.429	<0.001	Moderate
	Maximum length (mm)	0.148	0.048	Weak
	Stenosis (%)	0.439	<0.001	Moderate
Deep WMH	Maximum thickness (mm)	0.322	<0.001	Moderate
	Maximum length (mm)	0.136	0.070	Weak
	Stenosis (%)	0.318	<0.001	Moderate
Subcortical WMH	Maximum thickness (mm)	0.282	<0.001	Weak
	Maximum length (mm)	0.105	0.159	Weak
	Stenosis (%)	0.304	<0.001	Moderate

|*r*| < 0.3 = weak correlation; 0.3 ≤ |*r*| < 0.7 = moderate correlation. All coefficients were positive unless otherwise stated.

As shown in the correlation heatmap (Figure 3), WMH subregions were highly interrelated (e.g., periventricular vs. juxtaventricular, *r* = 0.88), suggesting a coordinated anatomical distribution of white matter injury. Plaque thickness and stenosis degree also showed strong collinearity (*r* = 0.83), while their cross-domain correlations with WMH burden were moderate (*r* ≈ 0.38–0.44).

### 3.4 Relationship between calcification patterns and WMH volume

To explore the effect of plaque calcification morphology on WMH volume, a multivariate linear regression model was constructed (Table 4). After controlling for confounding variables, type 6 (rim sign-positive) calcification was significantly associated with larger WMH volume (*β* = 0.359, 95% CI: 0.067–0.451, *p* = 0.045).

**Table 4:** Multivariate linear regression analysis of calcification patterns and WMH volume.

Variable	*β*	95% CI (lower–upper)	*p*
Type 2 vs. Type 1	−0.070	−0.345–0.206	0.621
Type 3 vs. Type 1	0.066	−0.249–0.381	0.680
Type 4 vs. Type 1	0.134	−0.263–0.532	0.508
Type 5 vs. Type 1	−0.196	−0.516–0.124	0.232
Type 6 vs. Type 1	0.359	0.067–0.451	0.045
Age	0.022	0.011–0.033	<0.001
Hypertension	0.066	−0.152–0.284	0.552
Hyperhomocysteinemia	0.095	−0.157–0.347	0.462
Maximum plaque thickness (mm)	0.065	−0.047–0.177	0.257
Stenosis degree (%)	0.009	−0.000–0.017	0.059

Other calcification types (types 2–5) did not show significant associations (*p* > 0.05). Among covariates, age was positively correlated with WMH volume (*β* = 0.022, 95% CI: 0.011–0.035, *p* < 0.001), while the association with stenosis rate approached but did not reach statistical significance (*p* = 0.059).

## 4 Discussion

This study demonstrated that carotid plaque morphology, particularly maximum plaque thickness and stenosis, was independently associated with the severity of white matter hyperintensity (WMH) in patients with carotid atherosclerosis. These associations remained robust after multivariate adjustment for vascular risk factors and lipid parameters. Moreover, the correlations were strongest in periventricular and juxtaventricular regions, suggesting a region-specific vulnerability of white matter to carotid atherosclerotic burden. Notably, rim sign-positive calcified plaques (type 6) were significantly related to higher WMH volume, indicating that specific calcification morphologies may reflect distinct pathophysiologic mechanisms linking large-artery atherosclerosis to small vessel disease.

Recent evidence increasingly supports the notion that large- and small-vessel pathologies are not independent entities but may share overlapping vascular risk factors and pathophysiologic processes (1, 7, 13). Both aging and hypertension contribute to endothelial dysfunction, vascular stiffness, and impaired cerebral autoregulation, thereby predisposing the brain to chronic hypoperfusion and WMH formation. Although diabetes and hypertension have been reported to synergistically increase the risk of micro- and macrovascular complications (15), the present study found no significant difference in diabetes prevalence between WMH groups, suggesting that structural vascular factors—rather than metabolic status alone—may drive the observed WMH burden.

Our findings are consistent with Shen et al. (2), who reported that extracranial carotid plaque characteristics, particularly calcification features, were independently associated with WMH severity. By combining CTA-based plaque quantification and automated MRI analysis, the present study strengthens the evidence that carotid atherosclerotic burden contributes to white matter injury. Furthermore, plaques with a rim sign-positive pattern were correlated with greater WMH volume. While calcification has traditionally been regarded as a marker of plaque stability, emerging histopathologic studies suggest that the distribution and morphology of calcification may have different implications: rim sign-positive plaques often correspond to complex inflammatory remodeling processes and perivascular fibrosis (16, 17).

We observed that plaque thickness and stenosis correlated most strongly with periventricular and juxtaventricular white matter WMH. These regions are supplied by long, end-arterial perforators and are located in watershed-like zones with limited collateral support, rendering them particularly susceptible to chronic hypoperfusion and impaired cerebral autoregulation (18–20). Thick plaques and higher-grade stenosis may reduce distal perfusion pressure, leading to subtle but sustained ischemic injury that preferentially affects periventricular and juxtaventricular white matter. This finding supports a hemodynamic mechanism in which carotid narrowing contributes to diffuse white matter damage.

Additionally, unstable atherosclerotic plaques may release microemboli, occluding penetrating arterioles and leading to scattered microinfarcts and localized gliosis, which subsequently manifest as confluent WMH on MRI (16). Chronic systemic inflammation and endothelial activation associated with atherosclerosis can further exacerbate blood–brain barrier disruption, contributing to white matter demyelination and rarefaction (17, 21). Taken together, these mechanisms suggest that carotid atherosclerosis can influence CSVD progression through both hemodynamic insufficiency and embolic injury.

Unlike plaque thickness and stenosis, plaque length was not independently associated with WMH severity in this cohort. This contrasts with several ultrasound-based studies that found significant associations between plaque length and both WMH burden and cardiovascular outcomes (10, 22–24). One possible explanation is the methodological difference: CTA offers higher sensitivity for detecting calcification but may underestimate soft plaque extent compared with ultrasound. Additionally, differences in patient selection (hospitalized vs. community populations) may account for the discrepancy. Nonetheless, the weak yet significant correlations between plaque length and periventricular/juxtaventricular WMH volumes suggest that elongated plaques could still influence local hemodynamics and cerebral blood flow patterns, thereby indirectly contributing to CSVD.

Interestingly, patients with severe WMH exhibited lower serum triglyceride and LDL-C levels, as well as a lower prevalence of hyperlipidemia. This paradox likely reflects confounding by lipid-lowering therapy. Older patients, who had a higher WMH burden, were also more likely to receive long-term statin treatment for secondary cardiovascular prevention. Statins effectively reduce LDL-C and TG but do not necessarily reverse established atherosclerotic plaque morphology or WMH progression. This finding underscores that improved lipid metrics may not directly translate into reduced CSVD burden once structural vascular damage has occurred. More broadly, CSVD has been linked to systemic vascular risk factors such as hypertension in prior studies (9, 13, 25).

The strengths of this study include the combined use of CTA and MRI data for comprehensive vascular and parenchymal assessment, as well as AI-assisted quantitative segmentation of WMH, which enhances measurement precision and reproducibility. Moreover, analyzing regional WMH volumes allowed us to identify spatially specific correlations that might be obscured in whole-brain analyses. In addition, previous work has suggested associations between CSVD and other systemic/organ conditions (26, 27), supporting the concept of multisystem vascular contributions.

This study has several limitations. First, the cross-sectional design precludes causal inference between carotid atherosclerosis and WMH progression. Second, although AI-assisted WMH segmentation improves efficiency and reproducibility, potential misclassification of perivascular spaces or lacunes cannot be completely excluded, which may lead to overestimation of WMH burden and introduce measurement bias. Third, the sample size was relatively modest and derived from a single-center inpatient cohort. Furthermore, the study population consisted predominantly of Asian hospitalized patients with a high prevalence of both carotid atherosclerosis and CSVD, which may limit the generalizability of the findings to other ethnic groups or community-based populations.

From a clinical standpoint, carotid plaque thickness and stenosis—readily quantifiable via CTA—may serve as useful imaging markers for identifying individuals at high risk of CSVD and cognitive decline, even in the absence of overt stroke or transient ischemic attack (TIA). Furthermore, recognition of specific calcification morphologies such as rim sign-positive plaques may provide additional insight into systemic vascular vulnerability and neurovascular coupling. Incorporating CTA-derived plaque metrics into existing MRI-based CSVD risk models may therefore improve early risk stratification and guide timely therapeutic interventions.

## 5 Conclusion

Carotid plaque thickness and stenosis are independent determinants of WMH severity, predominantly affecting periventricular and juxtaventricular regions. Rim sign-positive calcified plaques further indicate increased WMH burden, underscoring a potential mechanistic link between carotid atherosclerosis and cerebral small vessel disease. These findings highlight the importance of comprehensive vascular imaging in identifying high-risk patients and provide novel insights into the macro-microvascular continuum underlying cerebral white matter injury.

## Data Availability

The original contributions presented in the study are included in the article/supplementary material, further inquiries can be directed to the corresponding author.
